# Novel Telehealth Adaptations for Evidence-Based Outpatient Suicide Treatment: Feasibility and Effectiveness of the Crisis Care Program

**DOI:** 10.3390/healthcare11243158

**Published:** 2023-12-13

**Authors:** J. Conor O’Neill, Erin T. O’Callaghan, Scott Sullivan, Mirène Winsberg

**Affiliations:** Brightside Health, 2471a Peralta Street, Oakland, CA 94607, USAmimi.winsberg@brightside.com (M.W.)

**Keywords:** suicide intervention, telehealth, crisis intervention, outpatient, evidence-based treatment, CAMS

## Abstract

**Background:** Suicide rates in the United States have escalated dramatically over the past 20 years and remain a leading cause of death. Access to evidenced-based care is limited, and telehealth is well-positioned to offer novel care solutions. The Crisis Care program is a suicide-specific treatment program delivered within a national outpatient telehealth setting using a digitally adapted version of the Collaborative Assessment and Management of Suicidality (CAMS) as the framework of care. This study investigates the feasibility and preliminary effectiveness of Crisis Care as scalable suicide-specific treatment model. **Methods:** Patient engagement, symptom reduction, and care outcomes were examined among a cohort of patients (*n* = 130) over 16 weeks. The feasibility of implementation was assessed through patient engagement. Clinical outcomes were measured with PHQ-9, GAD-7, and the CAMS SSF-4 rating scales. **Results:** Over 85% of enrolled patients were approved for Crisis Care at intake, and 83% went on to complete at least four sessions (the minimum required to graduate). All patient subgroups experienced declines in depressive symptoms, anxiety symptoms, suicidal ideation frequency, and suicide-specific risk factors. **Conclusions:** Results support the feasibility and preliminary effectiveness of Crisis Care as a suicide-specific care solution that can be delivered within a stepped-care model in an outpatient telehealth setting.

## 1. Introduction

Suicide is a public health crisis and is the second leading cause of death among adults aged 18–45 in the United States [1]. Despite ongoing national suicide prevention and intervention initiatives, estimates suggest approximately 50,000 people (14.3 per 100,000) in the United States died by suicide in 2022, representing the highest national rate of suicide in decades [2]. Trends in suicide within the United States are alarming, with a 37% increase in suicide rates from 2000–2018, followed by a slight decline (−5%) from 2019–2020 [3]. Unfortunately, suicide rates have risen significantly in 2021 and 2022 post-COVID-19 pandemic (CDC, 2023), consistent with evidence suggesting that suicide rates may decline during disasters but increase in the period that follows [4]. Given the substantial societal and individual impacts of the COVID-19 pandemic within the past three years, feasible and effective treatments for suicide risk are needed. However, social perceptions of suicide can further exacerbate risk with affected individuals contributing to increased isolation and limited engagement with societal structures that can help improve access to essential care [5]. Shifting to an ecological understanding of suicide risk to emphasize comprehensive prevention methods within health systems and existing social structures is critical.

Further exacerbating this crisis in the United States, the proportion of individuals who experience suicidal thinking is 200 times higher than those who die by suicide and serves as a primary treatment target for healthcare systems. In 2021, 1.7 million adults made a suicide attempt, 3.5 million adults made a plan to attempt suicide, and 12.3 million adults reported serious suicidal ideation [2]. Contemporary suicide theories and empirical evidence suggest ideation-to-action pathways; that is, individuals who think about suicide are more likely to engage in suicidal behavior, with suicide attempt history serving as one of the most salient risk factors for death by suicide [6,7,8,9]. With over 12 million adults contemplating suicide, treatments that can effectively screen, detect, assess, and manage suicidal thinking are paramount to interrupt the ideation-to-action pathway and reduce suicide deaths. 

With such significant needs across a spectrum of suicide risk, stepped care models that offer treatment options proportionate to risk in settings with fewer restrictions are indicated [10]. Still, many individuals are evaluated through emergency departments when presenting with suicide risk [11]. While such settings serve a vital role in stabilizing the imminent risk of self-harm, hospitals are generally not designed to provide ongoing treatment of suicide risk [10]. Rather, hospitals are tasked with connecting patients to outpatient treatment that can effectively address suicidality [10]. This transition in care is critical and has been well established as a particularly vulnerable and high-risk time period [12,13]. Data show that in the month following discharge from inpatient hospitalization, suicide death rates are 200 times higher than those of the general public, and a heightened risk of suicidal behavior and death can persist for up to three months [14,15]. Yet, patients are often lost to follow-up during this period. Further, estimates suggest approximately one-third of patients fail to connect to an outpatient appointment within 30 days of discharge [16].

Access and connection to care is limited for individuals at risk for suicide, with a recent meta-analysis suggesting that fewer than 26% of individuals who died by suicide had contact with inpatient or outpatient mental health services within the year prior to their death [17,18]. Barriers to accessing mental healthcare services disproportionately impact certain populations, such as those in rural areas and minority populations [19,20]. To mitigate such barriers and enhance access within a stepped-care model, digital interventions and telehealth treatments have been identified as valuable care options [19,21,22]. Increasing care options serves as an important component of comprehensive suicide prevention and may reduce potential deaths [23]. By increasing access to care for those who truly need it, health systems may provide lifesaving care while reducing access gaps for vulnerable populations and deliver evidence-based care in a timely manner [24,25].

Telehealth treatment provides several benefits that can support access to care, including flexibility in scheduling, time savings and convenience, and geographical flexibility [26]. Evidence suggests that patients are more likely to attend their appointments virtually [27,28] with equivalent effectiveness in psychotherapy outcomes compared with in-person treatment in both outpatient and intensive outpatient levels of care [28,29]. However, there remains a dearth of telehealth suicide treatment options with notable hesitancy among clinicians in providing such specialty care in a telehealth setting [30]. 

Additionally, there are relatively few evidence-based approaches for suicide specific treatment (Dialectical Behavioral Therapy, Cognitive Therapy for Suicide Prevention, and the Collaborative Assessment and Management of Suicidality), none of which were developed specifically for telehealth delivery [31,32,33,34,35]. Despite these challenges, evidence for the effective treatment of suicide risk through telehealth settings is emerging.

The Collaborative Assessment and Management of Suicidality (CAMS) has demonstrated robust evidence in the treatment of suicide risk over the past several decades, especially with regard to reducing suicidal ideation to promote safety [36]. The CAMS framework adopts a collaborative, patient-centered approach that is empathic, therapeutic, and sensitive to unique suicide risk factors within each patient. During each session, CAMS utilizes the Suicide Status Form-4 (SSF-4) to guide a collaborative, therapeutic risk assessment, followed by the development and implementation of a suicide specific, individualized treatment plan that includes stabilization planning (e.g., a safety plan) [34]. CAMS is intended to be short-term, lasting approximately 6–12 sessions, and can be effectively implemented as a stepped-care option to treat suicide risk within outpatient settings [37].

CAMS has been studied in seven randomized controlled trials (RCTs), and in a recent meta-analysis, CAMS was found to have outperformed alternative interventions in significantly reducing suicidal ideation and depression and increasing hope [36,38,39,40,41,42,43,44]. Patients are more likely to remain in care and report better satisfaction in care compared with treatment as usual [36].

CAMS has also successfully been adapted for telehealth use across a range of settings and is being evaluated in multiple RCTs as a result of necessary conversions from in-person care during the COVID-19 pandemic [45,46,47,48]. However, to date, there have been no published studies demonstrating the feasibility and effectiveness of telehealth adaptations to CAMS in a diverse cohort of outpatient behavioral health patients. 

This study aimed to evaluate the feasibility and preliminary effectiveness of the Crisis Care program; a digitally adapted version of the CAMS framework for patients presenting with elevated suicide risk within a national outpatient telehealth treatment setting. 

Crisis Care seeks to provide timely access to treatment, increase access to care, and effectively treat patients with intermediate suicide risk who do not require immediate hospitalization, but do require suicide specific specialty care. Situated within a comprehensive tele-mental health treatment framework, Crisis Care offers continuity of care with step-down services and care coordination support for escalations to higher levels of care. The goals of the present study are as follows:

1. Examine time to treat, patient engagement, retention and response to treatment for patients with intermediate suicide risk within a telehealth outpatient setting.
2. Investigate preliminary clinical outcomes specific to suicide risk and mental health functioning.
3. Explore continuity of care outcomes for patients post discharge from Crisis Care.

The findings of this investigation provide valuable contributions to the field and demonstrate a promising telehealth care model to treat those at elevated risk for suicide. 

## 2. Materials and Methods

### 2.1. Participants

Participants who were enrolled in Crisis Care and completed their initial session during an 8-month period (October 2022–June 2023) were included in the study. Eligible participants included existing patients receiving care at a national telehealth company who were referred and enrolled in Crisis Care, as well as new patients who were enrolled in Crisis Care when initiating treatment with the telehealth company. 

Existing patients were enrolled in Crisis Care if they presented with elevated suicide risk, such as the endorsement of intense or persistent suicidal ideation, suicidal ideation with significant risk or precipitating event(s), suicidal thinking with a degree of planning or intent, or recent suicidal behavior (e.g., attempt). New patients were enrolled in Crisis Care if they reported suicidal ideation with a degree of planning endorsed through item 9 of the PHQ-9 or if they reported a suicide attempt within the past 12 months. Exclusionary criteria included active psychosis or mania, active primary substance use disorder diagnosis, active primary eating disorder diagnosis, or individuals who did not present with suicide specific treatment needs. Crisis Care is not a substitute for emergency care, and individuals reporting imminent risk of self-harm were referred to emergency services and not included in the present study. 

The sample (n = 130) included adults ages 18+ (m = 31, SD = 10.3) and was diverse in educational attainment and income (see Table 1). The majority of patients were female (58.46%), primarily diagnosed with major depression (66.92%), and included geographical diversity across 25 different states in the US. Approximately 42% (n = 54) of participants were new patients who enrolled in Crisis Care when initially signing up for services with the telehealth company, while 58% of participants (n = 76) were existing patients of the telehealth company who were referred internally to Crisis Care. 

### 2.2. Procedures

The study was approved by the WCG Institutional Review Board. A 16-week cohort was established to monitor patient engagement and response to treatment over time. Consistent with the CAMS model, Crisis Care does not have concrete requirements for length of care, as such decisions are informed by response to treatment and states of patient risk. Still, Crisis Care is intended to be completed within approximately 12 sessions. Therefore, a 16-week time frame was examined for all patients, with the expectation that the majority of patients would complete treatment prior to the conclusion of the 16-week window.

### 2.3. Patient Status

Patient status was monitored throughout the course of treatment. Patients were enrolled after consenting to terms and scheduling their initial Crisis Care intake session. Patients were either accepted or declined from Crisis Care during the initial intake session. Patients were declined if they did not meet eligibility criteria or elected to not move forward with Crisis Care treatment and were provided with appropriate referrals for further care. Accepted patients scheduled their follow-up sessions and began Crisis Care treatment. 

The course of treatment was adherent to the CAMS model. Patients were encouraged to attend weekly Crisis Care appointments for approximately 12 sessions. Successful completion of Crisis Care was based on the CAMS resolution guidelines with specified, clinical criteria associated with safety, stability, and reductions in suicide risk. This included three consecutive ‘low overall suicide risk scores’, evidenced by self-report ratings on the SSF-4 (see below), the absence of suicidal behavior in the past week, and the management of suicidal thoughts in the past week (if applicable). Clinical discretion was also applied for graduation so that patients may meet graduate eligibility criteria but continue in Crisis Care until the therapist and patient agree on program completion at the appropriate time. Patients were eligible for graduation from Crisis Care in as few as 4 sessions based on these criteria. 

### 2.4. Adaptations to CAMS

The CAMS intervention was digitally adapted with fidelity and implemented within a national telehealth company’s care process model. A digital version of each CAMS form was developed with screen-sharing capabilities to support the collaborative treatment experience. Documentation and completed forms were made available for clinicians and patients to readily access. Additional digital mental health tools including clinical check-ins (i.e., PHQ-9, GAD-7), asynchronous messaging, clinical alerts and notifications, and dedicated on-call clinical supervisor support were incorporated throughout the course of treatment to allow for real-time detection, assessment, and response to patient suicide risk and clinical symptoms. 

### 2.5. Crisis Care Treatment Components

The CAMS framework was implemented via video-based telehealth treatment sessions. In CAMS, the Suicide Status Form-4 (SSF-4) is used to guide patients through the completion of a suicide risk self-assessment in partnership with the therapist, while the therapist helps the patient identify key drivers related to their suicide risk that are used to construct an individualized treatment plan. CAMS is flexible in that various treatment techniques can be implemented during treatment to address the corresponding suicide drivers. A stabilization plan is developed in the first session and reviewed continuously throughout treatment. The stabilization plan is comparable to other safety planning interventions and consists of coping techniques, means restriction measures, social contacts, and emergency resources that the patient can utilize to support safety and reduce the risk of acting on suicidal thoughts or feelings. Suicide risk, response to treatment, and stabilization planning are evaluated throughout care in each CAMS session using the SSF-4.

In the present study, all core components of the CAMS intervention using the SSF-4 were implemented in each Crisis Care session (i.e., collaborative risk assessment, suicide-specific treatment planning, stabilization planning). Crisis Care patients also received a psychiatric evaluation by a psychiatric provider. Patients were prompted to complete weekly asynchronous clinical check-ins (i.e., PHQ-9, GAD-7), attend weekly live video-based Crisis Care treatment sessions (60-min intake, 45-min follow-up sessions), and had access to their clinicians through asynchronous messaging. Patients were instructed to use messaging as a supportive resource to communicate with their clinicians and not as a form of text therapy or for emergencies. All Crisis Care therapists were trained in CAMS. Ongoing consultation and support from CAMS-trained clinical leadership with expertise in suicide risk assessment and intervention was available to Crisis Care clinicians. 

### 2.6. Measures

Clinical outcomes were measured using three self-report instruments. The PHQ-9 is a 9-item, 4-point Likert scale self-report measure of depression symptom severity within the past two weeks. The PHQ-9 includes a specific item related to the frequency of suicidal thoughts (item 9). If suicidal ideation is endorsed, a follow-up item that screens for suicide attempt planning is administered. In the PHQ-9, item 9 was used as one data point for assessing suicidal ideation frequency in the past two weeks, and an additional assessment of suicide risk was measured using components of the CAMS intervention outlined below. Higher scores on the PHQ-9 indicate increased symptom severity and frequency of symptoms. The PHQ-9 has strong reliability and validity, with 88% sensitivity and 88% specificity for major depressive disorder (MDD) [49].

The GAD-7 is a 7-item, four-point Likert scale self-report measure of Generalized Anxiety Disorder (GAD) symptoms within the past two weeks. Higher scores reflect increased levels of anxiety. The GAD-7 has strong psychometric properties, with 89% sensitivity and 82% specificity for GAD [50].

The SSF-4 from the CAMS intervention is a 6-item, 5-point Likert scale self-report assessment measure that includes five domains of suicide risk grounded in suicide theory (self-hatred, agitation, hopelessness, psychological pain, distress) and an overall suicide risk score. Higher scores suggest higher levels of related symptoms and increased risk. The SSF-4 demonstrates strong convergent and criterion–predictive validity and moderate test–retest reliability, which is expected given the nature of suicide risk [51,52,53].

Patients were prompted to complete the PHQ-9 and GAD-7 at baseline and throughout the course of treatment at weekly intervals. Patients completed the SSF-4 at the start of each Crisis Care session in accordance with the CAMS intervention model. 

### 2.7. Data Analysis

The feasibility of implementing Crisis Care across a diverse cohort of patients was evaluated using descriptive methods. The average time to treat was calculated by observing the length of time from enrollment to the program to completion of the first Crisis Care session. 

Of the patients who were accepted to Crisis Care after the initial session, patient engagement and response to care were evaluated to identify three distinct subgroups: (1) those who graduated, (2) those who exited the program without graduating, and (3) those who remained in Crisis Care at the conclusion of the 16 weeks. 

Specific clinical outcomes were examined across each of the three subgroups, including changes in PHQ-9 overall scores, PHQ-9 item 9 (suicidal ideation frequency), GAD-7 scores, and the six domains of the SSF-4 at baseline and last-completed rating in order to calculate the average change in respective scores over time. 

The average time to graduation was calculated by examining the frequency distributions of the number of sessions completed at the point of graduation and the average length of time in weeks to complete the sessions. Reasons for exiting the program prior to meeting graduation criteria were qualitatively identified through medical record review, and continuity of care post graduation was assessed by observing attendance to an outpatient appointment within 30 days following step-down from Crisis Care to ongoing treatment within the telehealth company. 

## 3. Results

**Figure 1:**
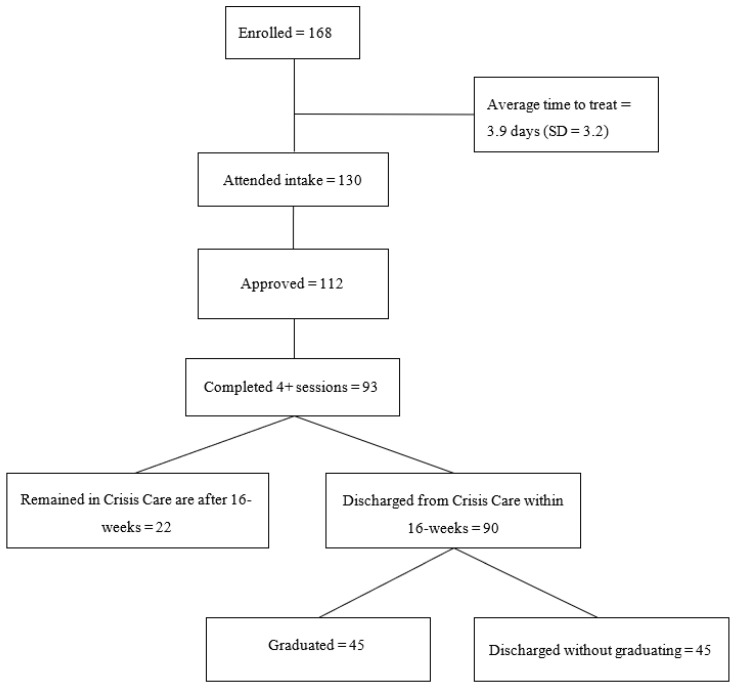
Crisis Care Patient Pathways.

A total of 168 patients consented to terms and enrolled in Crisis Care, 130 went on to complete their initial session, and 112 (86%) patients were approved to continue with Crisis Care treatment after the initial session. Of those approved, 93 (83%) patients completed at least four Crisis Care sessions. The average wait time for treatment from enrollment to attending the first session was 3.9 days (SD = 3.2) (Figure 1).

### 3.1. Graduated 

Of those approved for Crisis Care (n = 112), approximately 40% (n = 45) of patients graduated within a 16-week timeframe, and of those who attended at least 4 sessions (n = 93), 48% successfully graduated from the program in an average of 7.4 sessions. Thirty-one percent (n = 14) of patients graduated by the third follow-up session, which is the minimum number of sessions required to reach clinical graduation criteria. An additional 49% (n = 13) of patients graduated between four and eight sessions (m = 6.73) in an average of 8 weeks (SD = 3), and 20% (n = 9) graduated in nine or more sessions (m = 14.11) within 12 weeks on average (SD = 2). Taken together, over 50% (n = 23) of patients graduated by the fourth follow-up session (m = 4.07) within 6 weeks on average (SD = 3).

**Figure 2:**
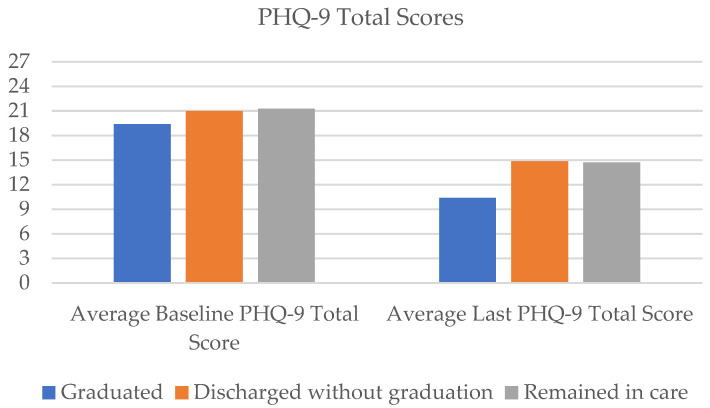
Average PHQ-9 total scores from baseline to last rating.

**Figure 3:**
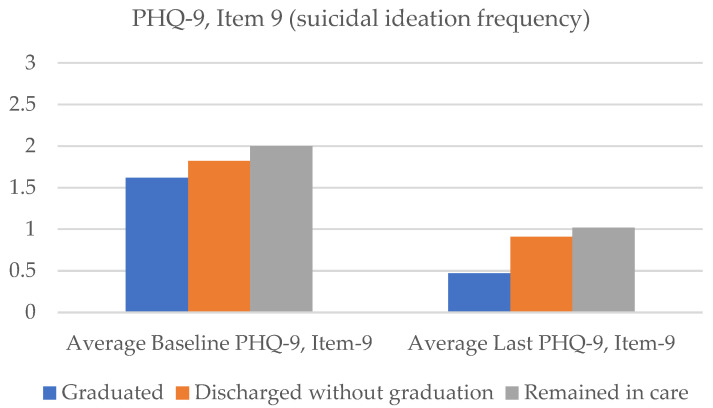
Average PHQ-9, item 9 (suicidal ideation frequency) from baseline to last rating.

**Figure 4:**
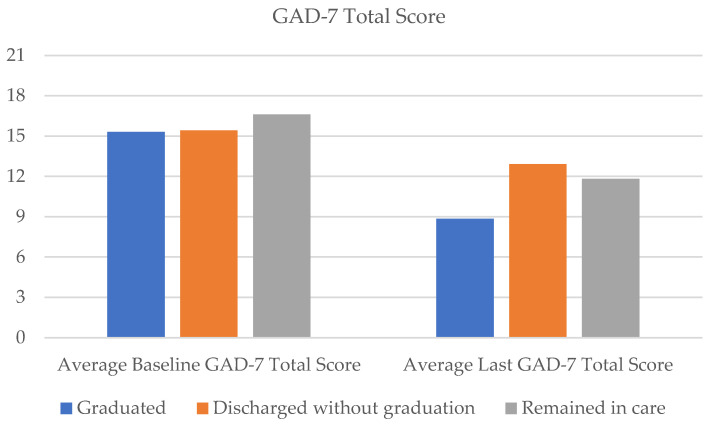
Average GAD-7 total scores from baseline to last rating.

Regarding clinical outcomes for all graduates (n = 45), the PHQ-9 total scores on average reduced by 46.5% from the baseline (m = 19.4, SD = 4.86) to the last score follow-up (m = 10.38, SD = 6.76) (Figure 2, Table 2). For suicidal ideation frequency, as measured by the PHQ-9 item 9, average scores reduced from 1.62 (SD = 0.96) at baseline to 0.47 (SD = 0.87), marking a 71.2% reduction in frequency ratings of suicidal thinking over the past two weeks (Figure 3). Average GAD-7 ratings reduced by 42.2% from the baseline (m = 15.31, SD = 4.49) to the last score (m = 8.84, SD = 6.18; Figure 4).

**Figure 5:**
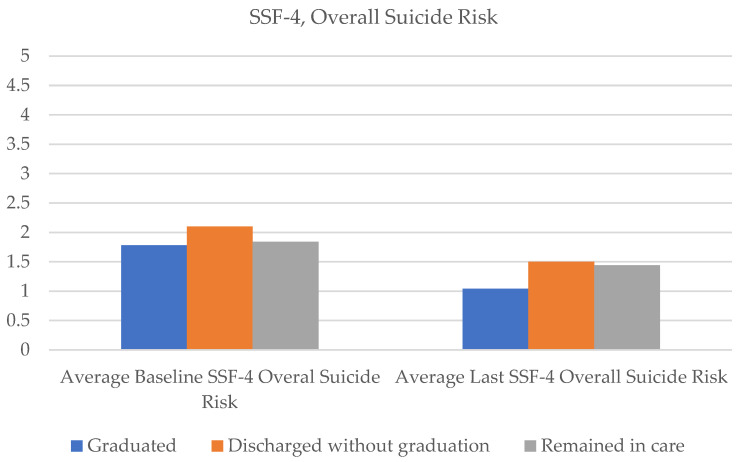
Average SSF-4, overall suicide risk scores from baseline to last rating.

Significant reductions were observed from baseline to program completion on all items of the SSF-4 among graduated patients. On average, self-reported agitation ratings reduced by 28.9%, hopelessness reduced by 48.4%, psychological pain decreased by 41.8%, self-hate declined by 42.5%, and stress decreased by 35.4%. Notably, overall suicide risk was reduced on average by 41.3% per patient self-report (Figure 5). Of the 45 patients that graduated, 100% stepped down to ongoing care within the telehealth company, and 87% (n = 39) attended at least one outpatient appointment within 30 days of completing Crisis Care.

### 3.2. Discharged without Graduation

Approximately 20% (n = 22) of patients who were approved for Crisis Care (n = 112) exited the program without graduating. The term “exit” refers to patients who left the program without achieving graduation status. Ten (45%) of those patients exited the program within the first three sessions. Reasons for exiting the program were mixed, with the majority of patients disengaging from treatment (60%, n = 13), 9% (n = 2) requiring a higher level of care, and 32% (n = 7) for other reasons (e.g., patient was appropriate for step-down prior to meeting graduation criteria or no longer consented to terms). 

Results suggest clinical symptom reduction even for patients who exited without graduating. On average, PHQ-9 total scores reduced by 29% (baseline m = 21.00, SD = 3.89; last score m = 14.86, SD = 7.85; Figure 2), item 9 of the PHQ-9 (suicidal ideation frequency) reduced by 50% (baseline m = 1.82, SD = 1.14; last score m = 0.91, SD = 1.15; Figure 3), and GAD total scores reduced by 16% (baseline m = 15.41, SD = 4.92; last score m = 12.91, SD = 6.73) from baseline to the last completed ratings (Figure 4). With regard to SSF-4 items, reductions were observed on average across all items from baseline to the last completed score. Agitation decreased by 17%, hopelessness by 43%, psychological pain by 38%, self-hate by 42%, stress by 31%, and overall suicide risk by 38% on average compared with baseline ratings (Figure 5). 

### 3.3. Remained in Crisis Care

Forty percent (n = 45) of approved patients (n = 112) remained in Crisis Care after 16 weeks. This subgroup of patients attended an average of 8.58 sessions over the 16-week period. However, nearly 70% (n = 31) of patients met the clinical criteria for graduation despite not having been formally processed for graduation from the program. Further, these patients experienced notable reductions in clinical symptoms. On average, total PHQ-9 scores declined by 31% from baseline (m = 21.29, SD = 4.12) to the last completed score (m = 14.73, SD = 7.35; Figure 2). The frequency of suicidal ideation declined by an average of 49% (baseline m = 2.00, SD = 1.02; last score m = 1.02, SD = 1.16) across the subgroup of patients (Figure 3), and GAD scores reduced by an average of 29% (baseline m = 16.6, SD = 3.74; last score m = 11.82, SD = 6.26; Figure 4). 

SSF-4 scores also declined, though to a lesser extent than those who exited the program (due to graduation or otherwise). On average, agitation was reduced by 11%, hopelessness by 21%, psychological pain by 18%, self-hate by 21%, stress by 13%, and overall suicide risk declined by 21% from baseline to the last completed Crisis Care session (Figure 5). 

Overall, results demonstrate that clinical symptoms, as measured by PHQ-9, GAD-7, and SSF-4 scores, improved from baseline to the last score in all Crisis Care clinical patient groups. 

## 4. Discussion

This study investigated the feasibility of implementing a novel telehealth solution for the treatment of elevated suicide risk within a cohort of outpatient behavioral health patients using a digitally adapted version of the CAMS intervention. Complementing the CAMS model with digital mental health tools and comprehensive care (e.g., med management, care coordination), Crisis Care was designed to deliver suicide specific treatment within a stepped-care model to quickly, safely, and effectively treat patients with escalated risk for suicide in the least restrictive setting within a scalable and sustainable framework. 

The results of the current study are encouraging with respect to the feasibility of implementation, preliminary effectiveness of clinical symptom reduction, and continuity of care. Patients at risk of suicide are at heightened risk when transitioning care [13] and should be seen within one week of referral [10]. Telehealth treatment has been identified as a viable option to create and support a fast connection to care [22]. In the present study, over 75% of enrolled patients attended their initial session, with an average time to treat of 4 days. These results support the use of telehealth treatment within a stepped-care framework as an effective solution to address key access gaps and expedite care connection for vulnerable populations at risk of suicide. 

While initial care connection and time-to-treat outcomes were promising, results related to engagement and adherence to treatment were mixed. The vast majority of patients (86%) who were seen for an initial Crisis Care evaluation were deemed appropriate for the program, suggesting accurate referral pathways. Over 80% of patients went on to complete at least four sessions, which is the minimum number needed for graduation, in addition to satisfying clinical criteria and clinician judgment. Less than half the sample graduated within the specified time period, and 80% of graduates completed treatment within eight sessions. This is consistent with the length of treatment completion rates established in the literature for the CAMS intervention [40] and suggests that digital adaptations of CAMS within a telehealth outpatient setting are feasible for a considerable portion of patients. Furthermore, all graduated patients stepped down to ongoing care at the telehealth company and attended at least one tele-behavioral healthcare visit within 30 days of discharge, supporting the advantages of a stepped-care model in providing continuity of care for those at risk of suicide. This is particularly encouraging given the risk of suicide remains particularly high for up to three months after a step-down transition in care [15]. By situating specialty suicide treatment within telehealth care models, health systems may close key gaps in care and support ongoing treatment to detect potential escalations of risk and respond accordingly in a least restrictive setting. 

Conversely, there was a decline in treatment engagement among a portion of patients within the first three sessions, and a quarter of patients who enrolled in Crisis Care did not move forward with completing the initial intake. It is unclear specifically why this portion of patients did not continue with treatment, and this warrants further examination. While creating quick access to care is critical, these results also suggest that additional efforts should target initial engagement in care at the point of referral and shortly after treatment commences. 

In addition, 40% of patients remained in treatment after 16 weeks, and 20% of patients were discharged before meeting graduation criteria. Upon further examination of these subgroups, it was clear that most patients were discharged due to disengagement, and those who remained in care without graduating attended an average of nine sessions over the 16-week timeframe, which is less than the recommended weekly session cadence. The finding that more than half of the patients who were discharged without formally graduating met the clinical graduation criteria set by CAMS, along with the 70% of those who remained in care after 16 weeks who also met the criteria, suggests that a majority of these patients indeed benefited from care and could explain reduced session attendance. Given that this study did not examine clinical outcomes beyond the 16-week timeframe, it is unknown whether this subgroup of patients ultimately graduated. Other possible explanations for patient disengagement could include patient-related factors such as time commitment challenges, preference for in-person care, limited acceptability of the treatment program, or treatment program related factors, such as difficulties accommodating preferred appointment times. However, reasons for patient disengagement were not overtly observable in the present study. Future research may support the identification of barriers to treatment adherence. Regardless of the reason, care coordination services and referrals were provided to all patients who either did not engage in Crisis Care or disengaged at any point in treatment. Given the portion of patients who experience difficulties adhering to care, it is recommended that care coordination services be included in specialty suicide treatment programs. 

Reductions in depressive symptoms, anxiety symptoms, suicidal ideation frequency, and suicide specific risk factors were measured for all patient subgroups (i.e., those who graduated, discharged without graduating and those who remained in care). These findings may be consistent with evidence that even single-session brief interventions to address suicide risk can be effective in reducing clinical symptoms and supporting safety [54].

This study was limited to a preliminary investigation of the feasibility and clinical effectiveness of the Crisis Care program. While efforts were made to track patient status, there was limited visibility as to why some patients discontinued care or were less engaged in treatment. Future studies focused on evaluating reasons for engagement in care will be beneficial. This study did not track clinical outcomes beyond the 16-week mark, and additional research evaluating the long-term results of the CAMS intervention administered within a stepped-care telehealth model should be explored. Finally, the clinical outcomes are preliminary and warrant further investigation using more rigorous study designs and analytic methods, such as a randomized controlled trial to determine the extent to which clinical symptom reduction is attributable solely to intervention components. However, only two patients in the study required referral to a higher level of care, which suggests that telehealth treatment of suicide risk in an outpatient setting is safe, feasible, and shows significant promise in effectiveness. Additional research to further evaluate the clinical effectiveness of suicide-specific telehealth treatment using inferential statistics is recommended to expand on these encouraging preliminary results. 

## 5. Conclusions

This work extends beyond and contributes to the existing literature in a number of ways. To our knowledge, this is the first study to examine the feasibility of treating a cohort of behavioral health patients using a telehealth-adapted version of CAMS within an established mental health services delivery model. Previous works have offered recommendations for telehealth adaptations to the CAMS framework [45,46,47,48], and the Crisis Care program has implemented these effectively within an established telehealth care model. In doing so, this study demonstrates feasible application of recommended adaptations to evidence-based treatment of suicide risk within a telehealth setting and offers a potential framework to scale such efforts and increase critical care options for people at risk of suicide. 

## Figures and Tables

**Table 1:** Demographics.

Characteristic	Study Sample (n = 130)
Age	m = 31.1 (SD = 10.3)
Sex	
Female	58.5%
Male	41.5%
Ethnicity	
White	62.3%
Hispanic/Latino	13.1%
Black	10.8%
Asian	3.1%
Other	9.2%
Not available	1.5%
Education	
<High School	3.9%
High School	46.9%
Associate’s Degree	16.2%
Bachelor’s Degree	24.6%
Advanced Degree	6.9%
Not available	1.5%
Annual Income	
<30 K	20.0%
30 K–60 K	29.2%
60 K–100 K	13.1%
<100 K	14.6%
Not available	23.1%
Geographic Region	
South	34.6%
Northeast	33.1%
West	24.6%
Midwest	7.7%

**Table 2:** Engagement and Clinical Outcomes.

Approved for Crisis Care (n = 112)	Graduated	Discharged without Graduation	Remained in Crisis Care
**Patient sample**	45 (40.2%)	22 (19.6%)	45 (40.2%)
Average # of sessions	7.4	4.3	8.6
Graduation criteria met	45 (100%)	12 (54.5%)	31 (68.9%)
**Clinical Outcomes**	**M (SD)**		
Baseline PHQ Total	19.4 (4.9)	21 (3.9)	21.29 (4.1)
Baseline PHQ-9, Item 9 (SI)	1.62 (0.96)	1.82 (1.14)	2 (1.02)
Baseline GAD Total	15.31 (4.5)	15.41 (4.9)	16.6 (3.7)
Last PHQ Total	10.38 (6.8)	14.86 (7.9)	14.73 (7.4)
Last PHQ-9, Item 9 (SI)	0.47 (0.87)	0.91 (1.15)	1.02 (1.16)
Last GAD Total	8.84 (6.2)	12.91 (6.7)	11.82 (6.3)
PHQ-9 Total % Diff Baseline vs. Last	−46.51%	−29.22%	−30.79%
PHQ-9, Item 9 % Diff Baseline vs. Last	−71.23%	−50.00%	−48.89%
GAD-7 Total % Diff Baseline vs. Last	−42.24%	−16.22%	−28.78%
**SSF-4**	**M (SD)**		
Baseline Agitation	3.16 (1.33)	3.25 (1.48)	3.05 (1.4)
Baseline Hopelessness	3.4 (1.25	3.65 (1.09)	3.51 (1.26)
Baseline Psychological Pain	3.13 (1.12)	3.67 (0.91)	3.62 (1.13)
Baseline Self Hate	3.24 (1.37)	3.8 (1.2)	3.47 (1.39)
Baseline Stress	3.96 (1.17)	4 (0.97)	3.73 (1.23)
Baseline Overall Risk of Suicide	1.78 (0.82)	2.1 (1.12)	1.84 (0.81)
Last rating—Agitation	2.24 (1.11)	2.82 (1.5)	2.71 (1.38)
Last rating—Hopelessness	1.76 (1.05)	2.73 (1.32)	2.76 (1.46)
Last rating—Psychological Pain	1.82 (0.96)	2.77 (1.34)	2.98 (1.37)
Last rating—Self-Hate	1.87 (1.04)	2.86 (1.55)	2.71 (1.42)
Last rating—Stress	2.56 (1.25)	3.23 (1.27)	3.24 (1.3)
Last rating—Overall Risk of Suicide	1.04 (1.01)	1.5 (1.01)	1.44 (0.66)
Agitation % Diff Baseline vs. Last	−28.87%	−17.02%	−10.85%
Hopelessness % Diff Baseline vs. Last	−48.37%	−43.14%	−20.55%
Psychological Pain % Diff Baseline vs. Last	−41.84%	−38.18%	−18.47%
Self-Hate % Diff Baseline vs. Last	−42.47%	−42.00%	−20.69%
Stress % Diff Baseline vs. Last	−35.39%	−31.48%	−13.46%
Overall Risk of Suicide % Diff Baseline vs. Last	−41.25%	−37.93%	−21.05%

## Data Availability

The datasets analyzed for the current study are available from the corresponding authors upon reasonable request.
